# H2valdien3 arrests the cell cycle and induces apoptosis of gastric cancer

**DOI:** 10.1515/med-2025-1208

**Published:** 2025-10-23

**Authors:** Chunyan Dang, Shuping Ma, Xuhui Zhao, Ruilin Wang, Ruimin Liu, Hongling Li

**Affiliations:** Department of Oncology, Gansu Provincial Hospital, Lanzhou, China; First College of Clinical Medicine, Gansu University of Chinese Medicine, Lanzhou, China; Department Five of Radiotherapy, Ganau Wuwei Tumor Hospital & Gansu Province, Wuwei Academy of Medical Sciences, Gansu Province, Wuwei City, 733000, China; Department of Oral and Maxillofacial Surgery, Gansu people's Hospital, 733000, Lanzhou, China

**Keywords:** H2valdien3, treatment, gastric cancer, apoptosis, cell cycle, β-catenin/c-myc

## Abstract

Despite significant advances in the diagnosis and treatment of gastric cancer (GC), its incidence and mortality remain high worldwide. Therefore, finding new anticancer drugs or treatment strategies for GC is crucial. The valdien ligand, which is not soluble in water, has demonstrated potential anticancer effects on cancer. This study highlights a water-soluble derivative of the H2valdien ligand derivatives, named H2valdien3, which can inhibit the proliferation of GC by arresting the cell cycle and inducing apoptosis. Initially, the inhibitory effects and cytotoxicity of H2valdien3 on the growth of AGS and MKN45 cells were evaluated using MTT assays. Microscopic observations revealed that H2valdien3-treated AGS and MKN45 cells exhibited deteriorated morphology. Hoechst fluorescent staining and flow cytometry results further demonstrated that H2valdien3 promotes apoptosis and arrests the cell cycle in GC cells. Additionally, the anticancer mechanism of H2valdien3 was found to involve the β-catenin/c-myc pathway. *In vivo* experiments showed that H2valdien3 inhibited GC proliferation without significantly affecting the weight of the mice. These findings suggest that the transformed H2valdien3 has potential anticancer properties with minimal side effects for GC treatment.

## 1 Introduction

Gastric cancer (GC) is a common malignancy originating from the gastric mucosal epithelium of the digestive tract. Globally, the morbidity and mortality of GC rank fifth and fourth, respectively [1,2]. Approximately 498,000 deaths annually are associated with GC, making it the second leading cause of cancer-related mortality in China, following lung cancer [3]. While surgery is widely used for primary GC treatment, the prognosis for patients with advanced GC remains poor [4]. Chemotherapy is crucial for treating advanced GC; however, due to chemotherapy resistance, the 5-year survival rate for GC patients is less than 30% [5]. Chemotherapy resistance is a major reason accounting for the treatment failure and short survival [6,7]. Therefore, the search for new therapeutic drugs is of extraordinary significance for improving the treatment and prognosis of GC.

H2valdien ligands are commonly used as organic synthesis reagents and liquid crystal materials [8,9]. They are multi-dentate ligands with various biological activities, including roles in fatty acid and amino acid metabolism, hematopoiesis, DNA synthesis, and anti-tumor effects [10,11]. Recent studies have focused on the anticancer properties of H2valdien ligands and their complexes. Previous research has shown that H2valdien, also known as N1N3-bis (3-methoxysalicyl) diethyl-trienamine, is a Schiff base with anti-tumor effects [12,13]. However, its bioavailability is limited due to its solubility only in dimethyl sulfoxide and not in water. To address this issue, a novel complex, H2valdien3, was synthesized by introducing two carboxyl groups (–COOH) to the H2valdien structure, enhancing its water solubility while retaining its anti-tumor effects. Our research group has long been committed to the value of H2valdien in inhibiting tumor cell growth by promoting tumor cell apoptosis. At present, we have found that water-soluble H2valdien can promote the apoptosis process of liver cancer cells, breast cancer cells, and colon cells and inhibit tumor growth [14–16]. In addition, it was also found to function in the liver protection, which demonstrates its special value for the treatment of cancer [17]. Through our investigation, we found the same effect in GC cells, which can induce apoptosis of GC cells; in addition, it can regulate the cell cycle and inhibit the proliferation process of GC.

The development and progression of GC are associated with various factors, including cell cycle regulation and apoptosis, which play critical roles in tumorigenesis. Apoptosis regulation involves complex processes, including the activation of apoptosis-related proteins such as BCL-2, BCL-XL, and BAD [18]. Additionally, the β-catenin/c-myc signaling pathway is activated in over 30% of GC cases, contributing significantly to the development of GC [19–21]. This study investigates the anticancer effects of H2valdien3 on cell cycle regulation and apoptosis-related proteins, including the β-catenin/c-myc signaling pathway in GC.

## 2 Materials and methods

### 2.1 Cell culture

The human GC cell line MKN45 and the human gastric adenocarcinoma cell line AGS were obtained from the Shanghai Institute of Biochemistry and Cell Biology, Chinese Academy of Sciences (Shanghai, China). MKN45 and AGS [22] were cultivated in RPMI 1640 medium (Yuanpei, Shanghai, China) supplemented with 10% bovine serum, 100 U/mL penicillin, and 100 μg/mL streptomycin in a humidified atmosphere with 5% CO_2_ at 37°C. Cells were used in all experiments during their logarithmic growth phase.

### 2.2 MTT assay

The inhibitory effects of H2valdien3 on cell proliferation were assessed using MTT assays. Single-cell suspensions were seeded in 96-well plates for 24 h and then treated with H2valdien3 at concentrations of 5, 10, 20, 30, and 40 mg/L. After incubation for 24, 48, and 72 h, 20 μL of MTT solution (MTT, Beyotime, Shanghai, China; 5 mg/mL) was added to each well. Subsequently, the supernatants were discarded, and 150 μL of dimethyl sulfoxide was added to each well. The absorbance at 490 nm (A490) was measured using a spectrophotometer. All experiments were conducted in triplicate.

### 2.3 Morphological observations

The effect of H2valdien3 on the morphology of MKN45 and AGS cells was observed using an inverted phase contrast microscope. Cells were seeded in six-well plates for 24 h and treated with H2valdien3 at concentrations of 5, 10, 20, 30, and 40 mg/L for 48 h. After incubation, the supernatant was removed, and cells were washed twice with PBS. Cells were then examined under an inverted phase contrast microscope.

### 2.4 Cell cycle analysis

To determine whether H2valdien3 inhibited cell cycles, flow cytometry was performed. Cells were seeded in six-well plates for 24 h and then treated with H2valdien3 for 48 h. After incubation, cells were collected, fixed with 70% ethanol at 4°C overnight, resuspended in 100 μg/mL RNase A (KeyGen Biotech, Nanjing, China), and stained with 50 μg/mL propidium iodide at 4°C for 30 min in the dark. All samples were analyzed using flow cytometry (BD Biosciences, America).

### 2.5 Hoechst fluorescent staining

Hoechst fluorescent staining was performed to detect apoptosis at the nuclear stage. Cells from exponentially growing cultures were seeded in 12-well culture plates and treated with H2valdien3 for 48 h. MKN45 and AGS cells were fixed in 4% paraformaldehyde and incubated at room temperature for 30 min. The fixative was removed, and cells were washed twice with PBS, resuspended in 50 μL of PBS containing 16 μg/mL Hoechst (Life Technologies, USA), and incubated for 10 min at room temperature. Samples were washed and examined using a Leica TCS-SP2 confocal microscope (Leica).

### 2.6 Flow cytometric analysis of apoptosis

Flow cytometry analysis was performed using the Annexin V-FITC/PI Apoptosis Detection Kit (KeyGEN, China). Cells were seeded in six-well plates and incubated for 24 h. After treatment with H2valdien3 for 48 h, cells were harvested and washed twice with cold PBS. Then, 5 μL of Annexin V-FITC and 5 μL of PI were added and incubated in the dark at room temperature for 15 min. The cell apoptosis rate was determined using flow cytometry and performed in triplicate.

### 2.7 Western blotting

Cells were treated with different concentrations of H2valdien3 for 48 h. For western blot analysis, cells were harvested, washed with cold PBS, and lysed in RIPA buffer (Beyotime, China). Protein concentrations were measured using the BCA protein assay reagent kit according to the manufacturer’s protocol. Thirty micrograms of protein were separated by 12% SDS-PAGE and transferred onto PVDF membranes. The PVDF membrane was blocked for 1 h with 5% skim milk and then incubated overnight at 4°C with suitably diluted primary antibodies. The membranes were incubated with horseradish peroxidase (HRP)-linked antibiotin and the appropriate secondary antibody in TBST with 0.5% BSA for 1 h at room temperature. Antibodies against β-catenin (ab16015), c-myc (ab32072), Cyclin B1 (ab32053), BCL-2 (ab182858), BCL-XL (32370), BAD (ab32445), Survivin (ab76424), and β-actin (8227) were purchased from Abcam Inc. Goat anti-rabbit or goat anti-mouse HRP-conjugated secondary antibodies were obtained from Abcam. Blots were detected using ECL, and protein bands were quantified by densitometric analysis using Image-Pro Plus software (Media Cybernetics, USA).

### 2.8 Mouse xenograft tumor model

Female BALB/c nude mice were purchased from Beijing Charles River Laboratory Animal Co., Ltd. The specific pathogen-free (SPF) nude mice were reared under sterile conditions at a constant temperature of 22–24°C and 50–55% humidity with a 12 h light/dark cycle. Mice were freely fed with standard laboratory forage and water. All mice were randomly divided into eight groups (*n* = 8): normal group, model control group, positive control group (5-FU group), and H2valdien3 groups (5, 10, 20, and 40 mg/kg). Cells were injected subcutaneously into the right front armpit of each nude mouse, except for the normal group, at a density of 1 × 10^8^ cells/mL. H2valdien3 was injected daily for 21 days. Mice in the 5-FU group were injected at a dose of 5 mg/kg every 3 days.

### 2.9 Mouse xenograft tumor model

Female BALB/c nude mice were purchased from Beijing Charles River Laboratory Animal Co., Ltd. The SPF nude mice were reared under sterile conditions at a constant temperature of 22–24°C and 50–55% humidity with a 12 h light/dark cycle. The mice were fed standard laboratory forage and water *ad libitum*. All mice were randomly divided into eight groups (*n* = 8): normal group, model control group, positive control group (5-FU group), and H2valdien3 groups (5, 10, 20, 40 mg/kg). Cells were injected subcutaneously into the right front armpit of each mouse, except for those in the normal group, at a density of 1 × 10^8^ cells/mL. H2valdien3 was injected daily for 21 days. Mice in the 5-FU group were injected at a dose of 5 mg/kg every 3 days for four times. The weight and volume of the tumors were recorded every 2 days. All mice were sacrificed 14 days after H2valdien3 treatment, and subcutaneous tumors were excised and frozen for further analysis. The tumor growth inhibition rate was calculated using the following formula: Inhibition rate (IR) (%) = (1 − Wt/Wc) × 100, where Wt and Wc represent the tumor weight of the treatment and control groups, respectively. Tumor volume (*V*) was calculated using the formula: *V* = (length × width^2^)/2.

### 2.10 Statistical analysis

Statistical analyses were conducted using GraphPad Prism 9. All data were presented as mean ± SD. One-way ANOVA and *t*-tests were used to determine statistical significance. Comparisons between two groups were performed using paired *t*-tests. A *P*-value < 0.05 was considered statistically significant.

## 3 Results

### 3.1 H2valdien3 inhibits GC proliferation through MTT assays

**Figure 1:**
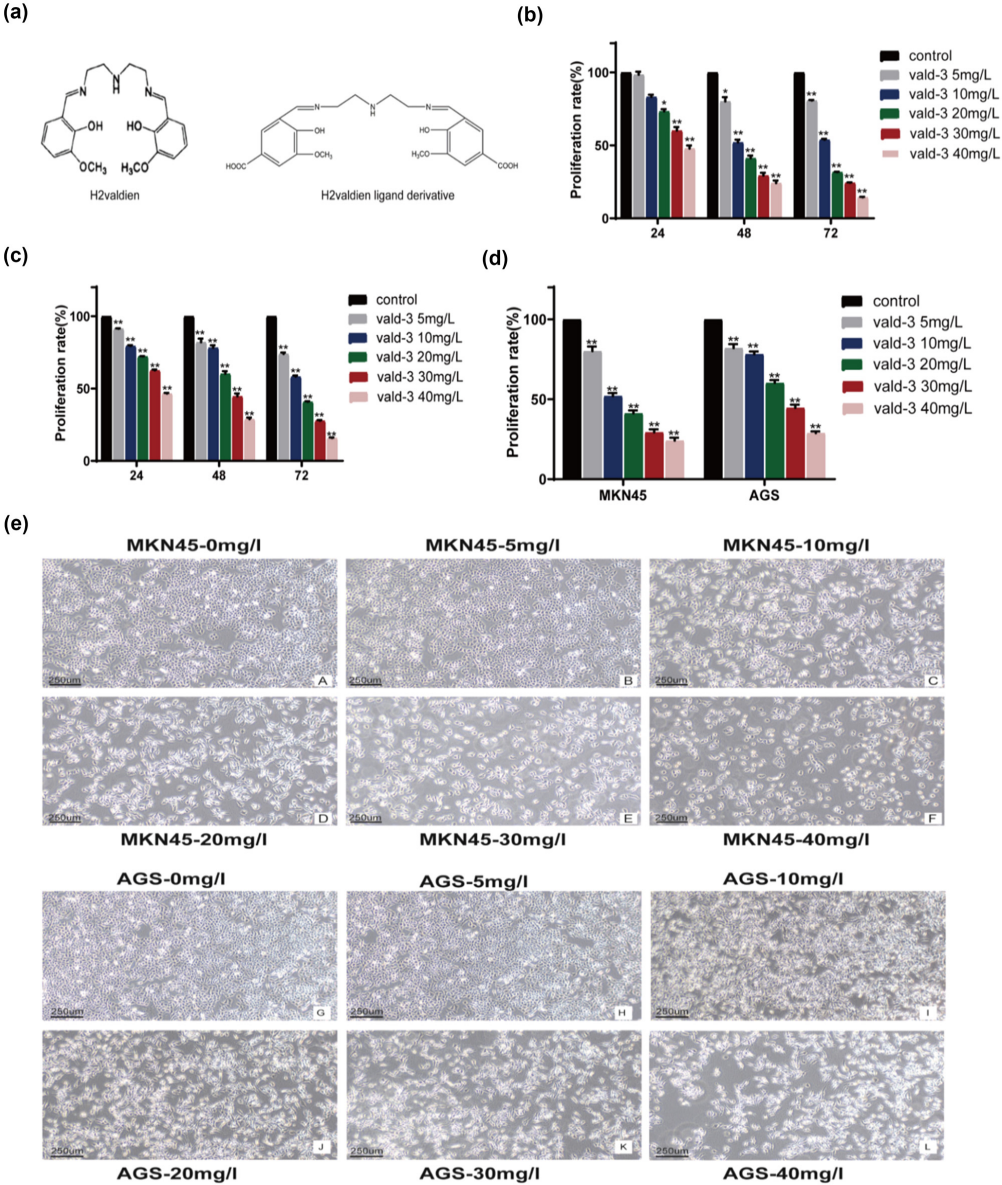
H2valdien3 inhibits GC proliferation through MTT assays. (a) Chemical structural formulas of H2valdien and H2valdien ligands. (b) H2valdien3 inhibits the proliferation of MKN45 in a time-dependent manner. (c) H2valdien3 inhibits the proliferation of AGS in a time-dependent manner. Cells were cultured in 96-well plates and treated with different concentrations of H2valdien3 (5–40 mg/L) for 24, 48, and 72 h, respectively. (d) H2valdien3 inhibits the proliferation of MKN45 and AGS in a concentration-dependent manner. Cells were cultured in 96-well plates and treated with different concentrations of H2valdien3 for 48 h. MTT assay was used to evaluate the proliferation rate. (e) Morphology of MKN45 and AGS cells after treatment with different concentrations of H2valdien3. Data are shown as mean ± SD of three independent experiments. *P < 0.05 or **P < 0.01 vs control.

To determine the proliferation rate of MKN45 and AGS cells treated with H2valdien3, MTT assays were conducted. MKN45 and AGS cells were treated with different concentrations of H2valdien3 (5, 10, 20, 30, and 40 mg/L) for 24, 48, and 72 h, respectively. The experimental group of MKN45 displayed significantly different proliferation inhibition rates compared with the control group in a time-dependent manner (Figure 1a). Similarly, AGS cells treated with H2valdien3 exhibited decreased proliferation in a time-dependent manner (Figure 1b and c). To investigate whether H2valdien3 inhibited the proliferation of MKN45 and AGS in a concentration-dependent manner, cells were treated with H2valdien3 for 48 h, resulting in significantly lower proliferation rates (Figure 1d). The results showed that different concentrations of H2valdien3 inhibited the proliferation of MKN45 and AGS in a dose- and time-dependent manner.

To observe the effect of H2valdien3 on the morphology of MKN45 and AGS cells, they were examined under an inverted phase contrast microscope. The blank control group of MKN45 had a polygonal shape and uniform size (Figure 1e). However, after H2valdien3 treatment, cells exhibited slower growth, deformed shapes, and irregular cell edges in a dose-dependent manner. Similarly, AGS cells treated with different concentrations of H2valdien3 showed deformed morphology compared with the control. These results indicated that H2valdien3 treatment deteriorated the morphology of MKN45 and AGS cells in a dose-dependent manner.

### 3.2 H2valdien3 arrests the cell cycle of MKN45 and AGS significantly

**Figure 2:**
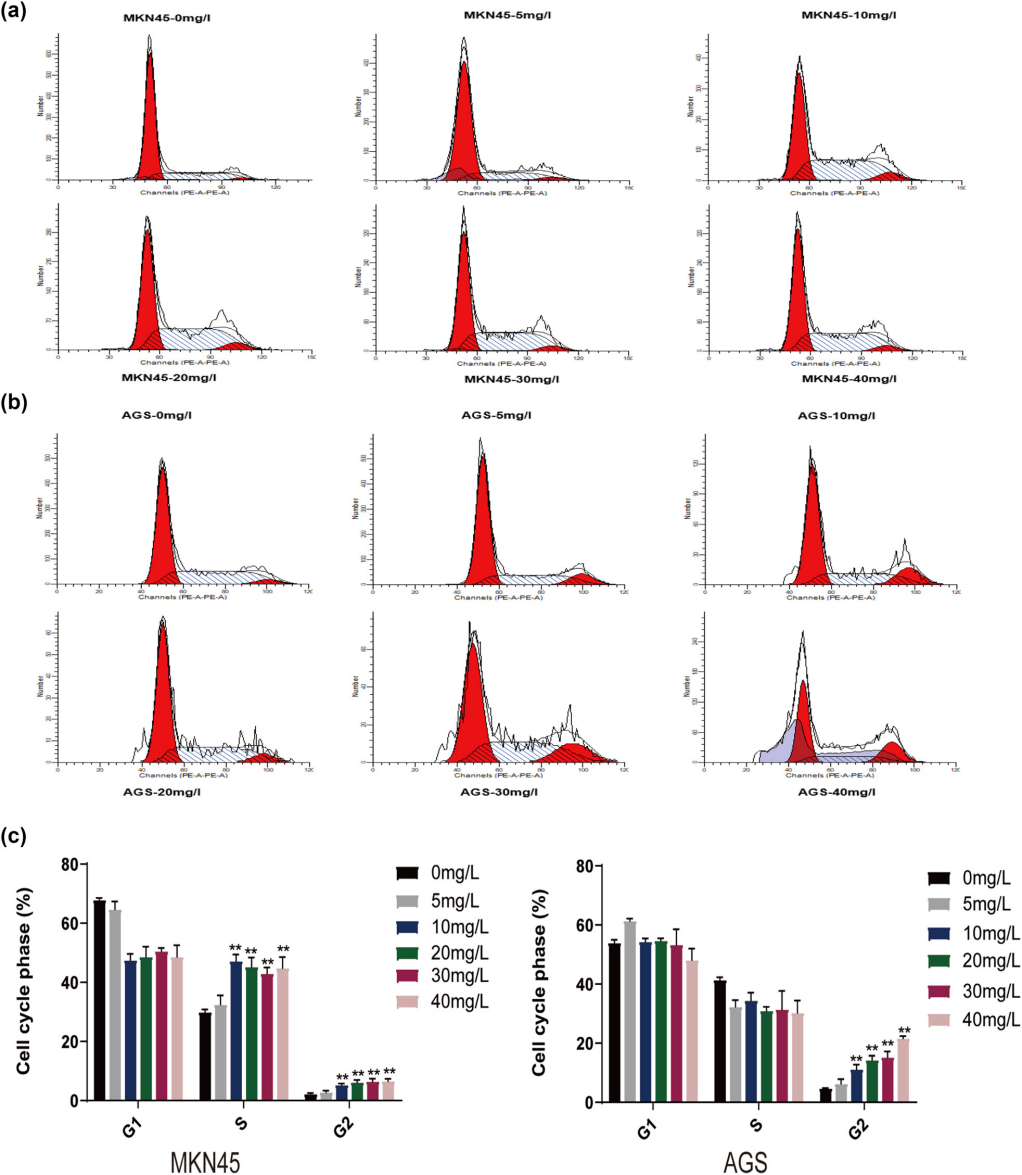
H2valdien3 arrests the cell cycle of MKN45 and AGS significantly. MKN45 and AGS cells were cultured in six-well plates for 24 h and then treated with different concentrations of H2valdien3 (5–40 mg/L) for 48 h. (a) Cell cycle stage of MKN45. (b) Cell cycle stage of AGS. (c) Effects of H2valdien3 on the cell cycle of MKN45 and AGS at various concentrations using flow cytometry. All results represent three independent experiments, and each value is mean ± SD. *P < 0.05 and **P < 0.01.

To examine the inhibition mechanism of H2valdien3 on the proliferation of MKN45 and AGS cells, different concentrations of H2valdien3 were used for 48 h, followed by flow cytometry analysis. MKN45 cells were arrested in the G2/S phase after H2valdien3 treatment, and the percentage of cells in G2/S phase increased significantly with higher H2valdien3 concentrations (Figure 2a) (*P* < 0.05). AGS cells were arrested in the G2 phase after 48 h of H2valdien3 treatment (Figure 2b), accompanied by a decrease in the G1 phase. The cell cycle phases of MKN45 and AGS cells treated with H2valdien3 are summarized in Figure 2c. These results indicated that the decreased cell proliferation of MKN45 and AGS cells treated with H2valdien3 was associated with cell cycle arrest.

### 3.3 H2valdien3 promotes apoptosis of GC cells

**Figure 3:**
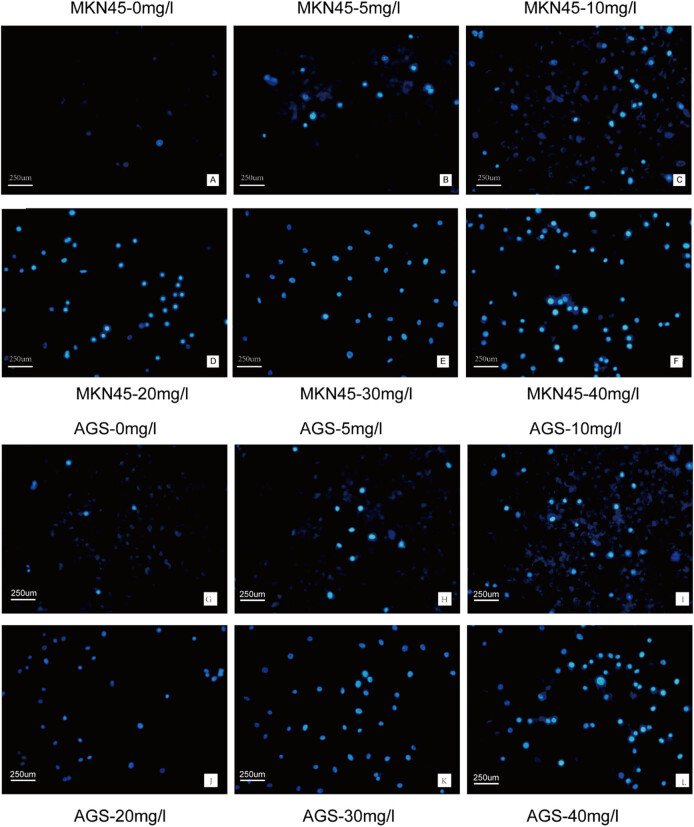
H2valdien3 induces apoptosis in MKN45 and AGS cells. MKN45 and AGS cells were stained with bisbenzimide trihydrochloride (Hoechst 33258) to evaluate apoptosis. (a)–(f) MKN45 cells and (g)–(l) AGS cells were cultured in six-well plates for 24 h and then treated with different concentrations of H2valdien3 (5–40 mg/L) for 48 h. The cells were observed under a microscope, and apoptosis increased with ascending drug concentrations.

**Figure 4:**
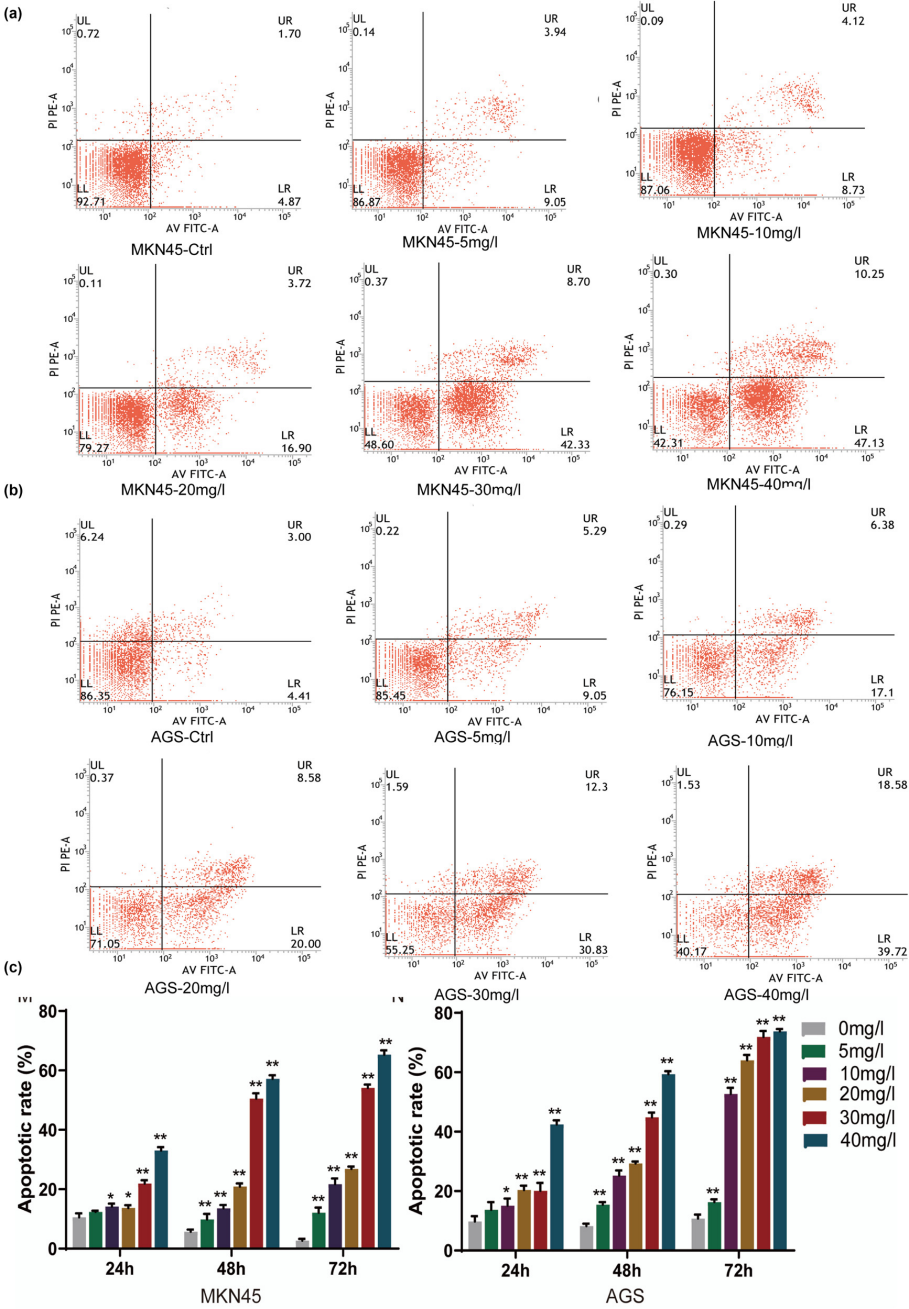
H2valdien3 promotes apoptosis of GC cells. (a) and (b) MKN45 and AGS cells were cultured in six-well plates for 24 h and then treated with different concentrations of H2valdien3 (5–40 mg/L) for 48 h. The Annexin V-APC/PI assay showed that the apoptosis rate of MKN45 and AGS increased in a concentration-dependent manner. (c) Quantitative analysis of the apoptosis rate of MKN45 and AGS. All results represent three independent experiments, and each value is mean ± SD. *P < 0.05 and **P < 0.01.

To examine whether H2valdien3 inhibited GC cells proliferation by inducing apoptosis, Hoechst staining and flow cytometry were performed. MKN45 cells treated with H2valdien3 showed chromatin condensation and the appearance of apoptotic bodies within 48 h (Figure 3a–f). Similarly, AGS cells exhibited significant apoptosis (Figure 3g–l). Flow cytometry combined with Annexin V-FITC/PI staining revealed that the apoptosis rate of MKN45 cells increased with increasing doses of H2valdien3 (Figure 4a). The apoptosis rate of AGS cells treated with H2valdien3 was significantly higher compared with the control group (Figure 4b). The apoptosis rates of MKN45 and AGS cells were calculated and displayed (Figure 4c). These results indicated that H2valdien3 treatment induced apoptosis of MKN45 and AGS cells in a time- and dose-dependent manner.

### 3.4 H2valdien3 arrests cell cycle and induces apoptosis via β-catenin/c-myc pathway in GC

**Figure 5:**
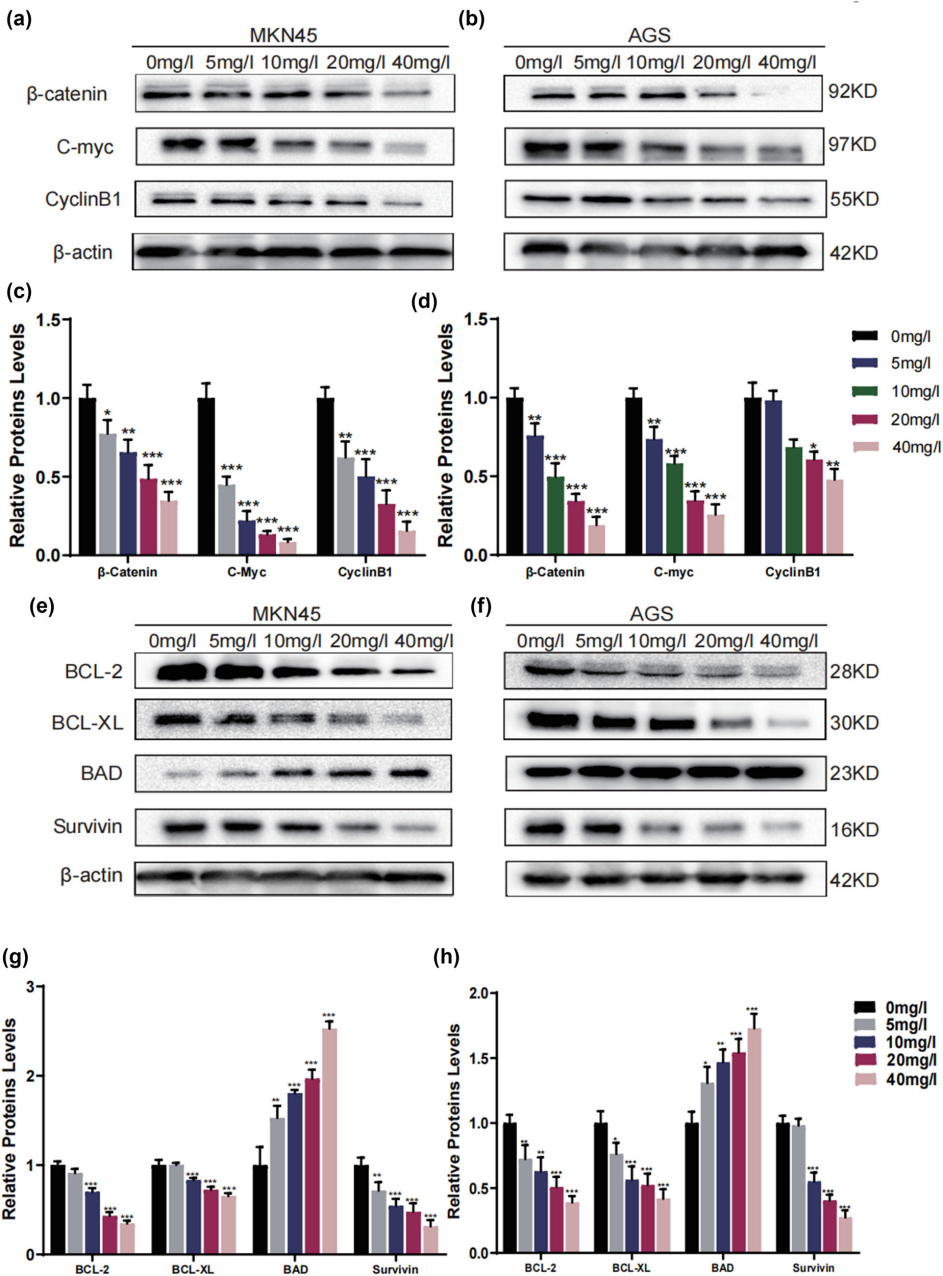
H2valdien3 arrests the cell cycle via the β-catenin/c-myc pathway and induces apoptosis in GC. (a) and (b) H2valdien3 decreased the expression of β-catenin, c-myc, and CyclinB1 in a dose-dependent manner in MKN45 and AGS. (c) and (d) Relative protein levels of β-catenin, c-myc, and CyclinB1 to β-actin in MKN45 and AGS. (e) and (f) H2valdien3 significantly decreased the expression of BCL-2, BCL-XL, and Survivin in a dose-dependent manner, while markedly increasing the expression of BAD in MKN45 and AGS. (g) and (h) Relative protein levels of BCL-2, BCL-XL, BAD, and Survivin to β-actin in MKN45 and AGS. Parallel blotting was performed with β-actin antibody. All results represent three independent experiments, and each value is mean ± SD. *P < 0.05 and **P < 0.01.

To demonstrate the potential mechanism of H2valdien3 in arresting the cell cycle and inducing apoptosis in GC cells, protein levels of cell cycle and apoptosis-related proteins were tested by western blotting. The protein levels related to the β-catenin/c-myc signaling pathway were detected after incubating cells with different concentrations of H2valdien3 for 48 h. The results indicated that H2valdien3 significantly inhibited the expression of β-catenin, c-myc, and CyclinB1 proteins in a dose-dependent manner (Figure 5a and b). Bar charts were constructed to summarize these data (Figure 5c and d). H2valdien3 significantly suppressed the expression of BCL-2 and BCL-XL in a concentration-dependent manner, while markedly increasing the levels of BAD. The results also showed that loading control level decreased when the drug concentration increased (Figure 5e and f). These results suggest that H2valdien3 arrests the cell cycle of MKN45 and AGS cells through the β-catenin/c-myc signaling pathway and induces apoptosis.

### 3.5 H2valdien3 inhibits the growth of GC cells *in vivo*

**Figure 6:**
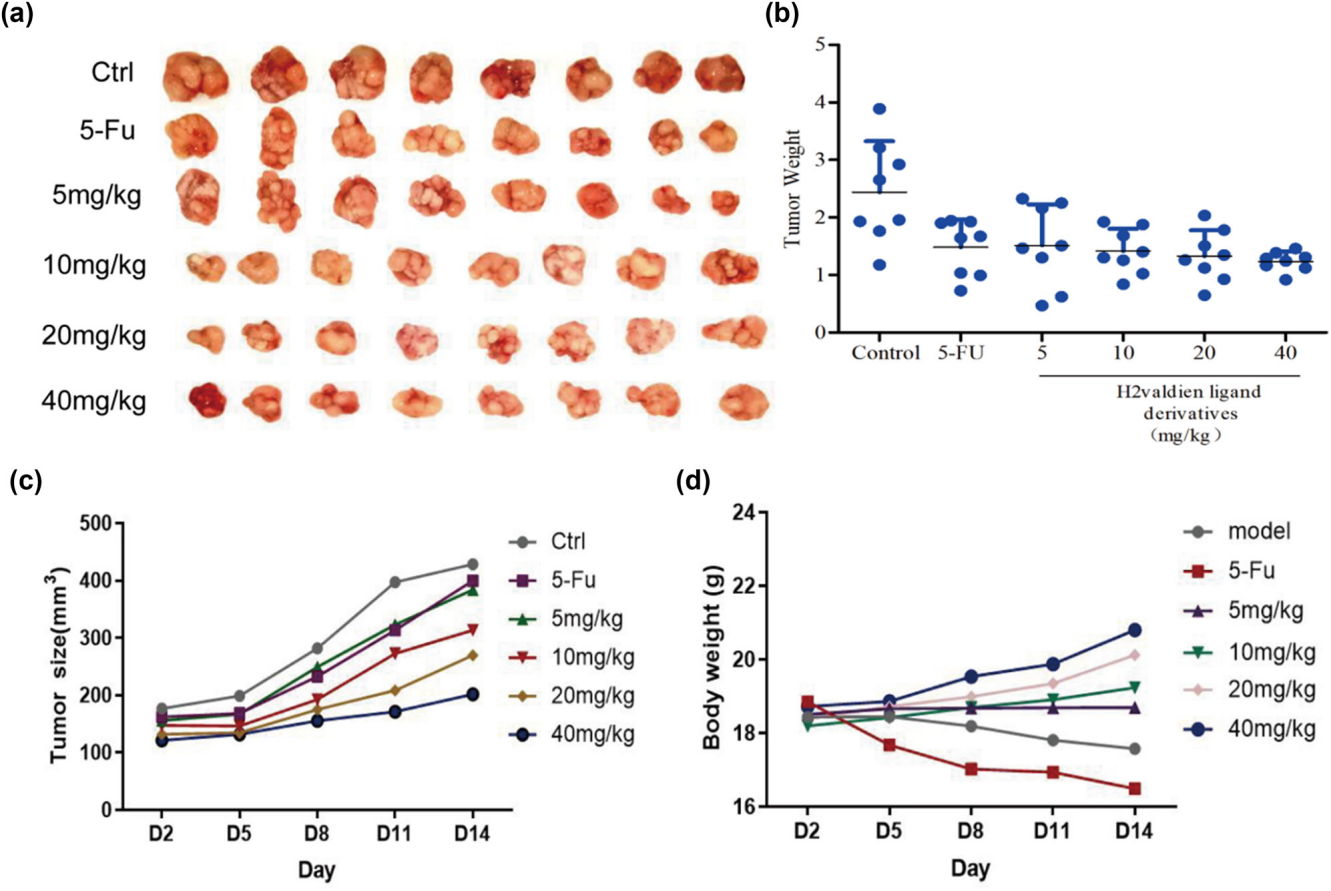
H2valdien3 inhibits the growth of GC cells *in vivo*. MKN45 cells were injected subcutaneously into nude mice. 5-FU and H2valdien3 (5–40 mg/kg) were injected as described in Section 2. (a) and (b) Tumor volumes increased in the control group and significantly decreased in the 5-FU and H2valdien3 groups. (c) Tumor volume and (d) body weight were measured every 3 days.

**Figure 7:**
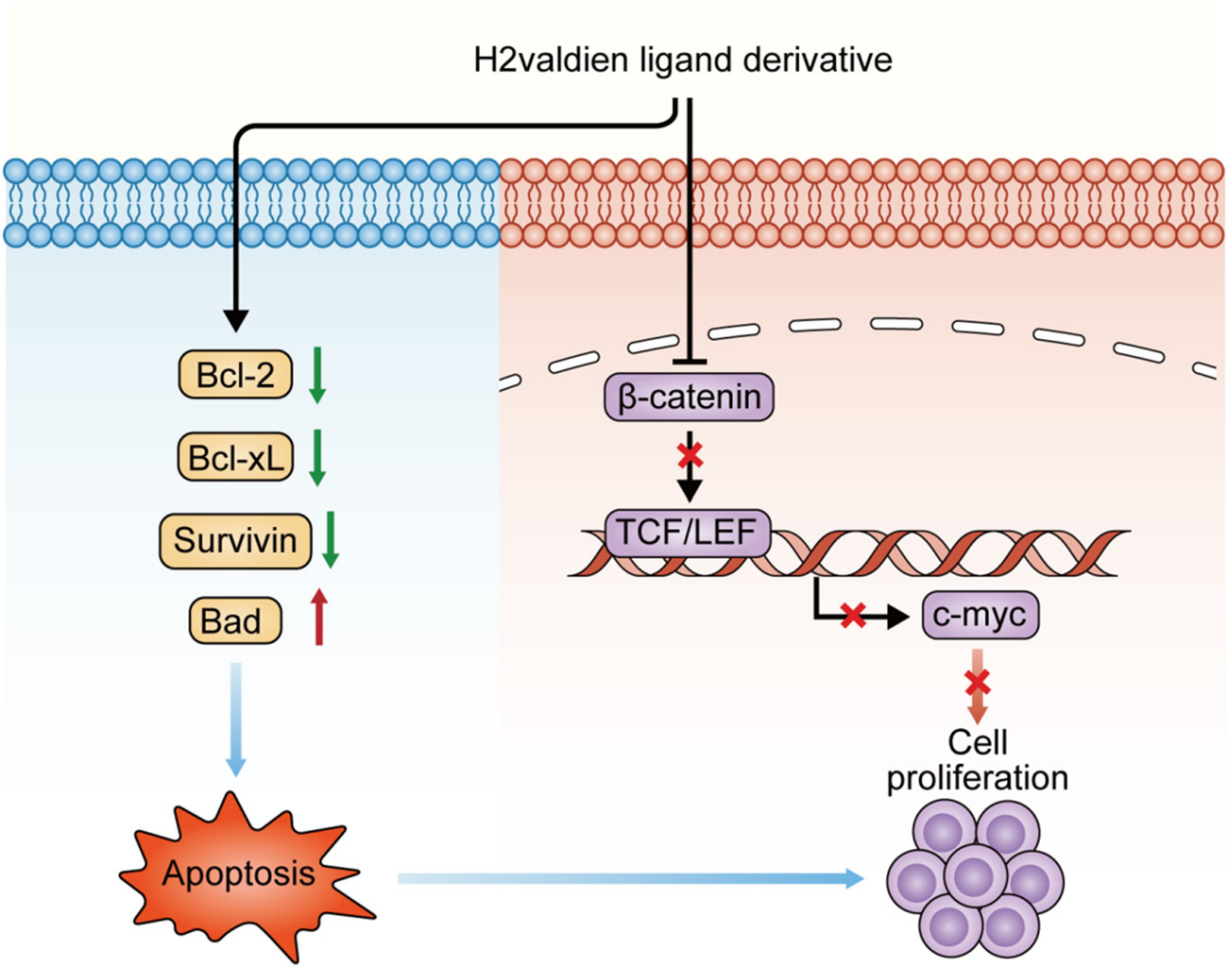
Regulatory mechanism of H2valdien3 arresting cell proliferation mainly via cell cycle inhibition and apoptosis related pathway.

To investigate whether H2valdien3 suppressed the growth of GC *in vivo*, MKN45 cells were injected into athymic BALB/c nude mice. Xenograft tumors treated with H2valdien3 grew more slowly compared with the control group (Figure 6a and b). The average tumor volume and weight in the H2valdien3 treatment group were significantly reduced (Figure 6c), indicating that H2valdien3 strongly inhibited tumor growth in the xenograft human GC model. While H2valdien3 shows promising dose-dependent effects *in vitro*, the complexities of *in vivo* pharmacokinetics, tumor microenvironment, drug stability, potential dose saturation, differences in model sensitivity, and the influence of the immune system might account for the lack of a dose-dependent effect on tumor growth *in vivo*. Moreover, there was no significant difference in body weight between the H2valdien3 treatment group and the control group (Figure 6d), suggesting low toxicity. The regulatory mechanism of H2valdien3 arresting cell proliferation, primarily via cell cycle inhibition and apoptosis-related pathways, is illustrated in Figure 7. These results suggest that H2valdien3 has potent anti-GC effects with low toxicity.

## 4 Discussion

GC is one of the most common malignant tumors globally. To date, the treatment of GC is based on accurate staging [23]. For patients with advanced GC, chemotherapy is crucial for improving survival rates [24]. However, various complex mechanisms often lead to chemotherapy resistance and subsequent treatment failure [25,26]. Therefore, there is an urgent need to discover new drugs with high efficacy and low toxicity for GC treatment. In this study, we investigated the anti-tumor effect and mechanism of H2valdien3 in GC. We found that H2valdien3 treatment inhibited GC cell proliferation, induced cell cycle arrest, and increased cell apoptosis. Additionally, H2valdien3 inhibited tumor growth *in vivo*.

Uncontrolled proliferation is closely related to tumorigenesis. This study demonstrated that H2valdien3 remarkably inhibited cell proliferation in MKN45 and AGS cell lines in both a concentration-dependent and time-dependent manner. Disruption of the cell cycle can inhibit cell proliferation and suppress tumor growth [27]. Our results showed that MKN45 cells were arrested in the G2/S phase, while AGS cells were arrested in the G2 phase. CyclinB1 is a key protein in the regulation of the G2 phase and plays a vital role in cell cycle regulation. CyclinB1 and CDK1 form a complex at the late stage of G2, thus regulating the transition from the G2 to the M phase [28]. Therefore, we speculate that H2valdien3 exerts antiproliferative effects by blocking the cell cycle transition from the G2 phase to the M phase.

Apoptosis, an independent physiological self-destructive process, involves the activation, expression, and regulation of a series of genes. There are two major apoptosis signaling pathways: the intrinsic mitochondrial pathway and the extrinsic death receptor-mediated pathway [29]. Bcl-2 family proteins play a crucial role in the intrinsic apoptosis pathway, including pro-apoptotic (Bax, Bad, Bok, Bak) and anti-apoptotic (Bcl-2, Bcl-w, Bcl-xL, Mcl1) members [30]. Bad promotes apoptosis by binding to the Bcl-2/Bcl-XL complex [31]. Our data showed that H2valdien3 treatment resulted in a dose-dependent increase in Bad levels and a decrease in Bcl-2 and Bcl-XL levels, suggesting that the regulation of Bcl-2 family proteins is associated with H2valdien3-induced apoptosis.

The β-catenin/c-myc signaling pathway is associated with cell proliferation, cell cycle regulation, and apoptosis in many cancers [32,33]. β-Catenin is the central molecule of the β-catenin/c-myc signaling pathway, and targeting β-catenin expression has beneficial anti-tumor effects [34]. C-myc is upregulated in about 30% of cancer cells and is associated with cancer progression [35]. CyclinB1 is the primary activator of cyclin-dependent kinase1 (CDK1) and, through complex formation with CDK1, controls cell cycle progression [36]. The activated β-catenin signaling pathway can also stimulate the transcription of CyclinB1 and c-myc, which are required for mitotic initiation [37]. Survivin, an important member of the apoptosis inhibitor family, has the strongest known inhibitory effect on apoptosis [38]. In this study, H2valdien3 significantly downregulated the expression of β-catenin, accompanied by decreases in c-myc, CyclinB1, and Survivin levels. These data indicate that the inactivation of the β-catenin/c-myc signaling pathway plays an important role in H2valdien3-induced GC cell death. Moreover, H2valdien3 effectively suppressed tumor growth *in vivo* without affecting the body weight of mice.

## 5 Conclusion

This study demonstrates that H2valdien3 can inhibit the proliferation of GC cells both *in vivo* and *in vitro*. The mechanism by which H2valdien3 arrests GC proliferation is likely regulated by the β-catenin/c-myc signaling pathway and apoptosis-related proteins. This study is significant as it explores the function and anticancer role of H2valdien3 in GC for the first time, suggesting that H2valdien3 can be a potential anti-tumor agent.
